# Editorial: Role of the antigen receptor in the pathogenesis of B-cell lymphoid malignancies

**DOI:** 10.3389/fonc.2023.1188375

**Published:** 2023-03-23

**Authors:** Andreas Agathangelidis, Manlio Ferrarini, Franco Fais

**Affiliations:** ^1^ Department of Biology, School of Science, National and Kapodistrian University of Athens, Athens, Greece; ^2^ Department of Experimental Medicine, University of Genoa, Genoa, Italy; ^3^ Molecular Pathology Unit, IRCCS Ospedale Policlinico San Martino, Genoa, Italy

**Keywords:** B-cell lymphoid malignancies, B cell receptor, immunoglobulin, chronic lymphocytic leukemia (CLL), immunogenetics, monoclonal B cell lymphocytosis (MBL), ontogenesis, familial CLL

This Research Topic contains nine manuscripts related to different scientific aspects of the *“Role of the Antigen Receptor in the Pathogenesis of B-Cell Lymphoid Malignancies”*. Each publication addresses pending issues, including (i) the role of the B cell receptor (BcR) in the ontogeny of these malignancies; (ii) the clinical relevance of particular properties of the clonotypic BcR immunoglobulin (IG) and other associated surface cell markers; and, finally, (iii) the benefits of targeting this crucial receptor for therapeutic purposes.

The notion that antigen selection of B cells through the BcR drives the pathogenesis of B-Cell Lymphoid Malignancies, such as CLL, is now well established. CLL is always preceded by monoclonal B-cell lymphocytosis (MBL), defined by a clonal B cell population of less than 5 × 10^9^/L and no symptoms or signs of disease. In this context, the review by Galigalidou et al. contains valuable information on the role of microenvironmental interactions in MBL ontogenesis and its progression to CLL. More specifically, the study of immune cell (B and T cells) receptor repertoires revealed important differences between MBL and CLL, alluding to distinct selection forces, both in terms of the nature of the selective antigens as well as the persistence of these interactions. Furthermore, the study of residual B cells revealed an impaired B cell production in the bone marrow, already at the stage of MBL. Hence, the tumor microenvironment in MBL may be pivotal for understanding the initial steps of malignant transformation.

Along the same lines, Kolijn et al. provided relevant information about the ontogenesis of a specific type of CLL, defined as familial CLL. Of interest, all four affected siblings of one of the families included in the study carried BcR IG expressing the IGLV3-21 gene with the hallmark R110 mutation. The BcR IG in 2/4 siblings were assigned to either stereotyped subset #2 or its immunogenetic relative subset #169, both of which belong to the clinically aggressive IGLV3-21^R110^ CLL subgroup. Furthermore, the CLL clones within each family exhibited driver gene mutations previously associated with IGHV mutational status, cytogenetic aberrations and stereotyped subsets. Altogether, these findings underline the notion that specific immunogenetic characteristics in combination with genetic aberrations drive CLL development, at least of the familial type.

In recent years, there has been accumulating evidence that the BcR expression levels and its functionality may associate directly or indirectly with other molecules. CD5 is considered among the most relevant ones in the context of CLL, which is located close to the BcR IG on the surface of the B cells and promotes cell survival and proliferation. In a brief research report, Maisano et al. described a novel peptide-based single-cell sorting methodology using the clonotypic BcR IG as bait. Hence, this study provided a proof of concept for the use of BcR IG ligands as probes for sorting and analyzing CLL clones. At the scientific level, transcriptomic analysis showed that the CD5 expression levels correlated with the expansion of the CLL clone, revealing a novel mechanism that could affect clonal expansion and persistence in CLL.

Over the years, immunogenetic studies in several B-Cell Lymphoid Malignancies support the theory for antigen drive by identifying distinct biases in the BcR IG gene repertoires. For example, the VH CDR3 sequences of the clonotypic BcR IG in CLL are characterized by length and amino acid composition restrictions. Rodriguez-Caballero et al. investigated the hydropathy index of the VH CDR3 in a large series of CLL patients and performed associations with other prognostic factors. Overall, two distinct subgroups of M-CLL patients emerged, displaying a neutral versus a negatively charged VH CDR3. Substantial differences were observed, with the M-CLL subgroup with neutral VH CDR3 being characterized by the predominance of the male gender, more advanced disease stage and a higher frequency of genetic aberrations, together with a higher rate of disease progression and shorter time-to-therapy (TTT). These findings further corroborate the relevance of the VH CDR3 in particular, and the BcR in general in CLL pathogenesis.

Another unique property of CLL is that a large fraction of clones (around 40%) are characterized by the expression of stereotyped BcR IG, which display distinct biological and clinical properties. Furthermore, CLL BcR IG have often been shown to carry autoreactive properties, alluding to a defect in immune tolerance in the respective patients. In two independent studies, Bagnara et al. and Vergani et al. performed high-throughput sequencing to explore the presence of stereotyped BcR IG in healthy donors. “CLL-like” stereotyped BcR IG were identified with no evidence of preferential accumulation in specific B-cell subpopulations (including CD5^+^ B cells at this pre-leukemic phase), possibly because either the level of autoreactivity is not high enough to be considered as dangerous by tolerance mechanisms or due to editing of the clonotypic IG light chain genes.

The mutational load of the rearranged IGHV gene is considered one of the most accurate prognostic markers in CLL; in detail, M-CLL patients have better outcomes than patients with U-CLL, probably because somatic IGHV mutations may affect the BcR IG structure towards abolishing polyreactivity. Kaufman et al. tried to address the latter by comparing cases with different ratios of replacement (R) mutations that lead to non-conservative amino acid changes (Rnc) to the combined numbers of conservative (Rc) and silent (S) amino acid mutations. When comparing time-to-first-treatment (TTFT) of patients with (S+Rc)/Rnc ≤ 1 and >1, TTFTs were quite similar. As the authors proposed, the structure of the BcR IG may not be the most critical factor for dictating outcomes in CLL, yet one should keep in mind that SHMs, even those of non-conservative nature, do not always affect the BcR IG structure substantially.

Over the years, significant progress has been made in the therapeutic management of CLL as well as other B-cell malignancies; in detail, targets in the BcR signaling pathway, such as BTK and PI3Kδ, have emerged as a successful treatment strategy. Unfortunately, a proportion of patients still relapse, indicating the need to identify new therapeutic targets. In this context, Sana et al. provided a comprehensive review regarding the importance of studying and identifying new potential druggable targets, focusing on NFAT. These transcription factors are involved in inflammation and the development of both autoimmune and neoplastic diseases. In more detail, NFAT1 and NFAT2 were described to affect cell proliferation and cell death after BcR stimulation. Finally, targeting NFAT was beneficial in treating CLL and lymphoma in preclinical models, with ABC DLBCL cells being particularly dependent on the activation of the NFAT pathway.

Of interest, Arbel et al. demonstrated that the BcR pathway can be efficiently targeted in CLL cells using proteolysis targeting chimeras (PROTACs). More specifically, the reversible non-covalent compound (NC-1) could degrade BTK in CLL cells, leading to decreased baseline BTK phosphorylation. Furthermore, this led to lower levels of activation of BTK and other signaling molecules downstream of the BcR pathway, following IgM engagement. These effects were also found in samples from CLL patients with clinical resistance to ibrutinib and the BTK mutation C481Y.

Overall, this collection contains several new information, concepts, and ideas related to the role of BCR in lymphoproliferative diseases that can also be used as further insights for work in this field.

## Author contributions

All authors listed have made a substantial, direct, and intellectual contribution to the work and approved it for publication.

